# Becoming active post-hospitalisation discharge – an exploration of motivational profiles during exercise change in obese patients

**DOI:** 10.1080/21642850.2018.1435998

**Published:** 2018-02-26

**Authors:** Anna Wasserkampf, Jens Kleinert, Chloé Chermette

**Affiliations:** Department of Health & Social Psychology, Institute of Psychology, German Sport University Cologne, Cologne, Germany

**Keywords:** Organismic integration theory, post-discharge, person-centred, self-determination, cluster analysis

## Abstract

Despite the urgent need to prevent weight regain in the long-term, it remains questionable whether inpatient multicomponent behavioural obesity treatments positively impact their patients, leaving them with favourable (i.e. autonomous) motivational profiles towards exercising. Based on Organismic Integration Theory, a sub-theory of Self-Determination Theory, this study retrospectively examined how exercise motivational profiles relate to exercise behaviour outcomes of a behavioural obesity treatment. Obese patients for whom outpatient treatment was deemed ineffective (*N* = 262; 34.2% female, body mass index >30 kg/m^2^) were administered to a 3-week inpatient obesity treatment. The study design incorporates both longitudinal and retrospective cross-sectional aspects. Patients completed questionnaires concerning exercise behaviour (pre-hospitalisation/6 months post-discharge) and behavioural regulations (6 months post-discharge). Exercise motivational profiles were generated based on the six behavioural regulations using *K*-means non-hierarchical cluster analysis. The self-reported dependent variable represents a change in patients’ exercise status (i.e. remaining inactive, becoming active). Chi-square tests related motivational profiles to exercise behaviour. Three profiles emerged: a *moderate-controlled cluster* (*n* = 80), a *moderate-autonomous cluster* (*n* = 78) and a *high-autonomous cluster* (*n* = 104). Of the patients who became active over time, the majority belonged to the high-autonomous cluster. No significant differences were found between patients who became active or remained inactive and whether they belonged to the moderate-controlled or moderate-autonomous cluster. Although the moderate-controlled and moderate-autonomous clusters differ greatly in their motivational quality, moderately controlled motivation does not seem detrimental regarding exercise change, as both clusters result in similar exercise behaviour outcomes.

## Background

In Germany, in cases where outpatient options are deemed ineffective and medical comorbidities necessitate rapid weight loss, obese patients (body-mass-index >30 kg/m^2^) are offered multicomponent three-weeks inpatient-obesity treatment aimed at changing dietary and exercise behaviours (Hauner et al., 2007). Multicomponent treatments are the first choice over surgical approaches (although the latter offers greater weight reduction than the former; Gloy et al., 2013), yet there is an outstanding need to investigate whether these inpatient multicomponent behavioural treatments positively impact patients, leaving them with favourable exercise behaviour change rationales and regulations (i.e. exercise motivational profiles), deemed promising regarding post-treatment exercise sustainment. Insights into obese patients’ exercise motivational profiles after an inpatient-treatment will contribute to our understanding of long-term exercise maintenance, as it reflects *whether* and *how* exercise has been internalised and for how long patients expend effort to sustain treatment outcomes.

Organismic integration theory (OIT), a sub-theory of self-determination theory (Deci & Ryan, 2000), represents a suitable theoretical framework with which to consider the multiple motivations people have for enacting (or failing to enact) intended behaviours like exercise. Within OIT, different regulatory forms of behaviours are aligned on a continuum of self-determination, which in order from left to right, range from: *Amotivation* characterises a condition in which individuals act without planning to attain an outcome (Deci & Ryan, 2004). *External regulation* describes a state in which people behave to obtain rewards, to comply with social pressures, or to evade punishment. *Introjected regulation* refers to behavioural engagement to obtain approval, praise, or to avoid feelings of socially induced guilt. *Identified regulation* represents a form in which people understand the value and significance of behaviour (Vansteenkiste, Niemiec, & Soenens, 2010). *Integrated regulation* describes participation in behaviour because it is consistent with other aspects of the self (Vansteenkiste et al., 2010). *Intrinsically motivated* behaviour refers to engagement that is undertaken for the enjoyment and satisfaction derived from the behaviour itself (Deci & Ryan, 2000).

Although the continuum of self-determination is central to OIT, its validity has recently been brought into question (Chemolli & Gagné, 2014). Concerns have arisen over (a) the need to recognise the multi- rather than unidimensionality of behavioural regulations (Chemolli & Gagné, 2014; Vallerand & Fortier, 1998), (b) the use of scoring protocols in which regulations are weighted and summed to build numerical indices (e.g. Relative Autonomy Index (RAI), Ryan & Connell, 1989), and (c) the need to consider the extent to which autonomous and controlled regulations are endorsed at similar (e.g. autonomous and controlled regulations are expressed to the same extent) or contrasting levels (e.g. autonomous regulations are higher than controlled regulations or v.v.; Brunet, Gunnell, Gaudreau, & Sabiston, 2015). While above studies collectively point into the same direction, a recent article by Sheldon, Osin, Gordeeva, Suchkov, and Sychev (2017) reaffirmed the validity of the continuum while arguing for an unweighted combined score as the most effective indicator of motivational quality. This conceptual mismatch in turn influences how behavioural regulations are assessed and scored.

While most OIT-researchers have so far examined exercise adherence via *variable-centred approaches* to analyse the impact of individual regulations on a given outcome (e.g. exercise), which is interesting, technically correct and accounts for motivation’s multidimensionality, variable-centred approaches still reject OIT’s central assumption of co-existing regulations when regulating behaviour (Friederichs, Bolman, Oenema, & Lechner, 2015; Vallerand, 1997). In contrast, *person-centred approaches* represent a suitable method to account for individual motivational constellations, and therefore their simultaneous impact on outcome parameters, as they examine how behavioural regulations are combined to form *motivational profiles* (Ratelle, Guay, Vallerand, Larose, & Senécal, 2007) and allow a more profound inspection of the underlying patterns of motivational processes compared to separate analyses of the six behavioural regulations (Guérin & Fortier, 2012; Vansteenkiste, Sierens, Soenens, Luyckx, & Lens, 2009). To date, studies using person-centred approaches have examined motivational profiles across a number of different samples (e.g. pupils (Hashim, Golok, & Ali, 2011), patient groups (Castonguay & Miquelon, 2017), athletes (Gillet, Vallerand, & Paty, 2013), the elderly (Stephan, Boiché, & Le Scanff, 2010) and settings (e.g. PE, (Ullrich-French & Cox, 2009), with no study, however, focusing on obese patients. The number of different profiles identified varies between three (Friederichs et al., 2015; Guérin & Fortier, 2012; Stephan et al., 2010), four (Castonguay & Miquelon, 2017; Matsumoto & Takenaka, 2004) or five clusters (Ullrich-French & Cox, 2009), with most studies confirming two opposing profiles: one showing high controlled and low autonomous regulation scores and the other showing high autonomous and low controlled regulation scores. Several studies also refer to profiles reflecting high or low levels of both controlled and autonomous regulations (i.e. combined profiles). Collectively, more *autonomous profiles* (i.e. high autonomous and low controlled regulation scores) are associated with more favourable exercise outcomes (e.g. exercise participation rate, enjoyment, maintenance and commitment Friederichs et al., 2015; Guérin & Fortier, 2012), compared to *controlled profiles* (i.e. high to moderate controlled and low autonomous regulation scores; Miquelon, Chamberland, & Castonguay, 2016). *Combined profiles* (i.e. high scores on both autonomous and controlled regulations) predict exercise, however with less intensity and length compared to autonomous profiles (Miquelon et al., 2016) and low chances to maintain exercise over time (Matsumoto & Takenaka, 2004). Overall, given that most studies identify three to five qualitatively different profiles, it seems inadequate to classify individuals as autonomous or controlled motivated regarding exercise (Guérin & Fortier, 2012) and, therefore, that a person-centred approach is warranted.

Despite the urgent need to prevent weight regain after behavioural treatment-induced weight loss, there is still a lack of research examining how much and for how long patients will expend efforts to sustain intervention-induced exercise levels. Given that motivation is clearly among the most promising candidates for achieving long-term weight loss, future research is warranted to unravel the motivational profiles of obese patients, particularly those who successfully maintain their activity level following therapy cessation. This information may provide useful insights for understanding if patients truly and permanently took on an intervention’s goal as their own; crucial information for the development of tailored interventions to prevent relapses, particularly amongst those patients who show less favourable motivational profiles towards exercising. Moreover, as integrated regulation might be the sole regulation that indicates how ‘*far*’ a patient is into the internalisation of exercise behaviour (i.e. how far an exercise-related outcomes have been brought into congruence with personally important values and goals; Deci & Ryan, 2004) as opposed to the remaining more static regulations, it was decided to include integrated regulation into analyses.

In order to contribute to the limited body of OIT-based literature concerning obese post-discharge patients, the present study aims (1) to retrospectively describe different exercise motivational profiles six months post-discharge and (2) to examine changes in exercise behaviour from pre-hospitalisation to six months post-discharge, in order to ascertain whether obese patients who were inactive pre-hospitalisation remained inactive or became active over time. (3) Additionally and although this study is exploratory in nature, it is expected (based previous empirical findings) that patients who became active over time reflect profiles high in autonomous motivation, while patients who remained inactive show profiles high in controlled regulations. The identification of a combined profile is also anticipated, although no specific hypotheses regarding its shape or relation to exercise will be proposed (as profiles in obese patients have never been investigated previously).

## Methods

### Participants

Obese patients are eligible for receiving inpatient multicomponent behavioural obesity treatment in Germany when outpatient treatment has been ineffective and medical comorbidities require rapid weight loss. Patients were recruited through physicians, dieticians and psychologists from six German clinics that offered inpatient obesity treatment. The study sample comprised 262 patients (*n* = 90 females, age *M* = 44.65, *SD* = 11.24, Table 1) that completed initial assessments (i.e. pre-hospitalisation) and entered the three-week inpatient obesity treatment.

**Table 1.** Patients characteristics pre-hospitalisation and at the end of the 6-months post-discharge phase.

	Total (*n* = 262)	Female (*n* = 90)	Male (*n* = 172)
Demographics			
Age (years)	46.53 ± 9.76	44.65 ± 11.24	47.5 ± 8.76
Higher education^a^	23.8	20.2	25.9
Marital status/living with partner	69.1	64	71.9
Body measurements			
Height (m)	1.75 ± .09	1.74 ± 9.5	1.75 ± 9.23
Weight (kg)	122.27 ± 22.7	112.98 ± 22.47	127.37 ± 21.27
Body mass index (kg/m^2^) - pre-hospitalisation	40.3 ± 8.8	37.2 ± 8.36	41.8 ± 8.63
Body mass index (kg/m^2^) - post-discharge	36.9 ± 10.11	33.2 ± 10.36	38.8 ± 9.47
Co-morbidity			
Hypertension	67.6	64.8	69
Diabetes mellitus	30.1	20.5	35.1
Dyslipidemia	21.2	10.2	26.9
Orthopedic complaints	64.7	69.3	62.4
Bronchial asthma	3.5	6.8	1.8
Sleeping problems	7.7	9.1	7
Psychological/psychiatric disorders	8.1	19.3	2.3
Exercise behaviour			
Weekly Leisure-Time Activity Score, pre-hospitalisation	16.3 ± 17.8	12.23 ± 14.85	18.25 ± 18.89
Weekly Leisure-Time Activity Score, post-hospitalisation	23.19 ± 17.81	20.94 ± 16.8	24.39 ± 18.34
Exercise change groups			
Remaining inactive	45.9	55.7	40.8
Becoming inactive	8.9	10.2	8.3
Remaining active	17	6.8	21.9
Becoming active	28.2	27.3	29

Note: Data are given as mean ± SD or %. Pre-hospitalisation = previous to the 3-weeks inpatient obesity treatment; post-discharge = at the end of 6-months follow-up period post-discharge.

^a^Higher education comprises vocational baccalaureate diploma, high school diploma, university degree.

### Study design

The study was set within the context of an inpatient obesity treatment, involving three-weeks of multicomponent behavioural therapy and a six-month follow-up phase without intervention post-discharge across all clinics. The study design incorporates both longitudinal and retrospective cross-sectional aspects. The longitudinal assessment was used to determine how patients changed their exercise behaviours from pre-hospitalisation to six months post-discharge. The retrospective cross-sectional measures were used to determine how differences in exercise status over time relate to exercise motivational profiles (i.e. the assessment of behavioural regulations for exercise post-discharge on one single occasion). The Ethics Committee of the university approved the study and patients provided signed informed consent prior to participation.

### Clinical treatment regimen

During the three-week hospitalisation, a multidisciplinary team of dieticians, physicians, psychologists and exercise instructors delivered a multicomponent intervention intended to achieve behavioural modifications, particularly by improving nutritional and exercise habits. The multicomponent interventions were similar (i.e. in terms of length and treatment content) across clinics (according to interdisciplinary guidelines for prevention and treatment of obesity of the German Diabetes Association, Hauner et al., 2007) and consisted of individual and/or group psychotherapy sessions targeting self-monitoring practices (e.g. to learn to control one’s weight, eating and exercise behaviours), relaxation and goal setting training, problem solving strategies (e.g. to overcome barriers), social support, and social competence training (i.e. self-assertion training; practicing capabilities necessary for a comfortable interaction with others in the patient’s nearby environment). Indoor and outdoor exercise therapy (e.g. walking, Nordic-walking, aqua gymnastics), dietetics and nutritional advice/consultations led by either trained physiotherapists or nutritionists.

### Measures

In addition to socio-demographic data (age, gender, education level, marital status), body measurements (i.e. height, weight) and comorbidities were assessed (Table 1).

#### Behavioural regulations

The German translation of the Behavioural Regulation in Sport Questionnaire (BRSQ; Kleinert & Pels, 2013; Lonsdale, Hodge, & Rose, 2008) assessed behavioural regulations for sport and exercise (it is important to note that in the German language no distinction is made between ‘sport’, ‘exercise’ or ‘physical activity’; ‘sport’ is used interchangeably for all kinds of movements ranging from jogging, gym workouts, yoga, and rehabilitation classes, thus explaining the selection of a sport and exercise instrument). Preliminary validations confirmed the instruments validity and reliability (Kleinert & Pels, 2013). Following the stem of ‘I engage in exercise or sport … ’, the scale’s 24-items were partitioned into six subscales (each containing four items) measuring patients’ behavioural regulations for sport and exercise: *amotivation* (e.g. ‘but I question why I continue’; *α* = 0.78), *external regulation* (e.g. ‘because if I don’t other people will not be pleased with me’; *α* = 0.93), *introjected regulation* (e.g. ‘because I would feel ashamed if I quit’; *α* = 0.85), *identified regulation* (e.g. ‘because it teaches me self-discipline’; *α *= 0.88), *integrated regulation* (e.g. ‘because it’s a part of who I am’; *α* = 0.92) and *intrinsic motivation* (e.g. ‘because I enjoy it’; *α* = 0.98). The questionnaire instructions asked participants to base responses on sport or exercise they had engaged in during the weeks prior to the end of the six-month post-discharge phase. Responses are made on a seven-point Likert scale ranging from 1 (not at all true) to 7 (very true).

#### Exercise behaviour

The German version of the Godin Leisure Time Exercise Questionnaire (Godin, 2011; Lippke, Fleig, Pomp, & Schwarzer, 2010) was used to assess participants’ self-reported bouts of mild (e.g. yoga, bowling), moderate (e.g. easy bicycling, fast walking) and strenuous (e.g. jogging, football) activity during a typical week, both pre-hospitalisation and at the end of the six-month post-discharge phase, in a retrospective manner. For each intensity, the total number of bouts was weighted by three, five or nine (representing metabolic equivalents) and summed, reflecting a weekly leisure-time activity score. According to the relationship between the volume of exercise and health benefits, three categories can be defined: 24 units or more represents being active and achieving substantial health benefits, 14 to 23 units represents being moderately active and achieving some health benefits, and less than 14 units represents being insufficiently active and achieving marginal benefits (Godin, 2011).

Upon assessment completion, exercise-change groups were classified according to Godin’s cut-off point of 24 units (including mild, moderate and strenuous intensities) and in line with exercise recommendations of overweight and obese patients (Murdy & Ehrman, 2013). Accordingly, patients who achieved less than 24 units were regarded as inactive (as scores below 24 units do not result in substantial health benefits and are below the recommended amount of exercise behaviour for obese patients; Godin, 2011; Murdy & Ehrman, 2013) and those who achieved more than 24 units were regarded as active. Consequently, four exercise change groups emerged from pre-hospitalisation to six-month post-discharge: (1) ‘*remaining inactive group’* (remaining below 24 units over time), (2) ‘*becoming inactive group*’ (dropping below 24 scores over time), (3) ‘*remaining active group’* (remaining above 24 units over time) and (4) ‘*becoming active group*’ (surpassing 24 units over time). As the overall goal of the treatment was to improve patients’ activity levels, comparisons were only made between those patients who were successful in becoming more active (i.e. becoming active group) and those patients who were unsuccessful (i.e. remaining inactive group). It should be stressed that the practice of dichotomising data from continuous scores into more discrete categories is not without criticism, as it can lead to unreliable results (Cohen, 1983). However, in this case, data were split into active and inactive patients as we were interested to see if patients engaged in sufficient amounts of exercise and achieved health benefits (i.e. scores above 24), rather than the exact amount of exercise they engaged in.

### Data analysis

All analyses were conducted using SPSS for Windows (Version 23). Data were inspected and cleaned accordingly. Preliminary assumption testing was conducted to check for normality, univariate and multivariate outliers. One individual was detected as an outlier and consequently removed from the data (based on Mahalanobis distance criteria Tabachnick & Fidell, 2014).

To answer the first research question, an iterative *K*-means non-hierarchical cluster analysis was employed to explore motivational profiles based on six behavioural regulation scores including the all four exercise change groups. The advantage of cluster analysis is that it follows a heterogeneous cohort of individuals and classifies them based on their similarity across specified variables, resulting in a smaller number of mutually exclusive clusters (Hair & Black, 2000). A double-split cross-validation procedure was applied to investigate the cluster solution stability (Friederichs et al., 2015; Vansteenkiste et al., 2009). The sample was split into two subsamples (A and B) and the *k*-means cluster procedure was applied to each subsample. The new cluster solutions were then compared for agreement with the original cluster solutions in both subsamples using Cohen’s kappa, in which a kappa of at least .6 was deemed acceptable (Friederichs et al., 2015; Vansteenkiste et al., 2009). The three cluster solution from the subsamples showed the highest Cohen’s kappa and consequently determined the definitive cluster number choice. Between-cluster differences in exercise regulations and exercise behaviour were assessed using Analyses of Variance (ANOVAs). To answer the second research question, the dichotomous definition that classifies patients with exercise scores above 24 units as active and those lower than 24 units as inactive was utilised. Specifically, exercise change groups’ pre-hospitalisation weekly leisure-time activity scores were compared with those assessed at the end of the six-month post-discharge. The final research objective was addressed by conducting multiple Chi-square tests to test for relationships between motivational profiles and exercise change groups.

For all ANOVAs that proved to be significant, Bonferroni post-hoc tests were carried out. Effect sizes and measures of strength (partial eta squared and Cramer’s *V*) were calculated for ANOVAs and Chi-square tests. Analyses were conducted using a significance level of .05.

### Ethics Statement

The study follows the principles of the Declaration of Helsinki.

## Results

### Participant characteristics

At the time of hospitalisation, the average patient weight was 122.27 kg (*SD* = 22.7; range 76.70–202.4 kg, BMI *M* = 40.3 kg/m^2^, *SD* = 8.8). On average, patients reported being overweight for 24.67 years (*SD* = 12.8) and had taken part in one or more therapy regimen (*M* = 1.15, *SD *= 1.42) prior to hospitalisation. Most patients engaged in exercise therapy (i.e. aqua gymnastic, indoor exercises in groups, individually in the gym) two to three times per week during their hospitalisation, a little more than a third received nutritional advice once a week while around 20% received individual and 30% received group psychotherapy on a weekly basis during their admission. Sample characteristics are depicted in Table 1.

### Exercise motivational profiles 6-months post-hospitalisation

**Figure 1.**
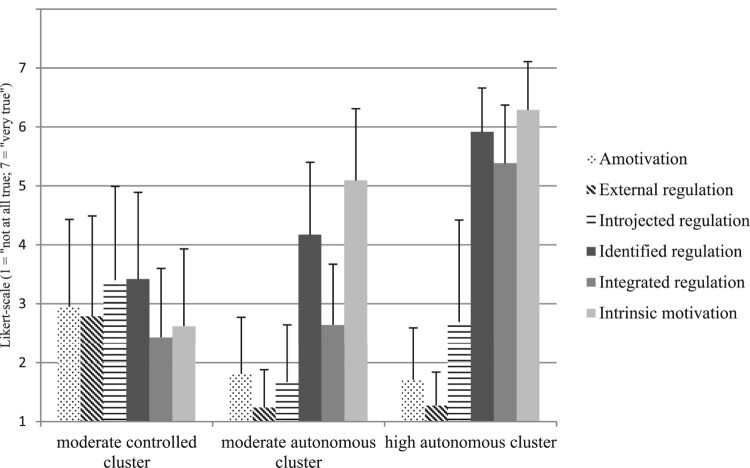
Motivational profiles based on behavioural regulations for exercise 6 months post-discharge (*M*, *SD*). Moderate-controlled cluster: *n* = 80; Moderate-autonomous cluster: *n* = 78; High-autonomous cluster: *n* = 104.

A two cluster solution was tested first, followed by a three, four and finally a five cluster solution. The validation procedure showed that the assortment of three clusters best represented the motivational profiles (see Figure 1). The first cluster, referred to as the *moderate-controlled cluster*, comprised 30.5% (*n* = 80) of the patients. Patients belonging to this cluster scored low on more autonomous forms of regulations and moderate on more controlled forms, with highest scores on introjected and identified regulation. The second cluster was referred to as the *moderate-autonomous cluster* and comprised 29.8% (*n* = 78) of the patients. Members of this cluster showed high scores on both identified regulation and intrinsic motivation but moderately low scores on integrated regulation, while scores on controlled regulations were low across amotivation, external and introjected regulation. The third cluster was referred to as the *high-autonomous cluster* and comprised 39.7% (*n* = 104) of the patients. Patients in this cluster showed high scores on the more autonomous types of regulations, intrinsic motivation in particular, while moderately high scores were found for introjected regulation and low scores for external regulation and amotivation.

### Changes in exercise behaviour from pre-hospitalisation to 6-months post-discharge

Pre-hospitalisation, patients had an average weekly leisure time activity score of 16 (*SD* = 17.8). At six months post-discharge, the activity score was 23 (*SD* = 17.81). When analyzing changes in exercise behaviour from pre-hospitalisation to the end of the six-month post-discharge, it was revealed that 46% of the patients remained inactive (i.e. the remaining inactive group), while 28% became active over the course of time (i.e. becoming active group), as seen in Table 1.

### Relationship between exercise motivational profiles and exercise change groups

Regarding the number of patients who became active over time, the majority belonged to the high-autonomous cluster (*n *= 38), the second most to the moderate controlled (*n *= 19) and the fewest to the moderate-autonomous cluster (*n *= 16; Table 2). In contrast, of the patients who remained inactive over time, the majority belonged to the moderate-controlled cluster (*n *= 53) and the second most to the moderate-autonomous cluster (*n *= 43).

**Table 2.** Differences between motivational profiles, socio-demographic variables, behavioural regulations and exercise change groups.

	Moderate-controlled cluster *n* = 80	Moderate-autonomous cluster *n* = 78	High-autonomous cluster *n* = 104	*F*/*x*^2^	*p*	Partial *η*^2^/*V*
Gender: % female	37.1	29.2	33.7	3.22		.11
Age	47.32 (8.42)	47.17 (9.54)	45.44 (10.81)	1.07		.01
Marital status/living with partner: %	29.8	27.1	43.1	3.23		.11
Education: % high education^a^	13.8	26.9	29.1	6.49	.039	.16
Body Mass Index	41.41 (7.94)	40.04 (6.33)	38.92 (5.94)	3.06	.049	.02
Amotivation	2.96 (1.49)^b,c^	1.81 (0.96)	1.71 (0.88)	32.29	<.001	.20
External regulation	2.74 (1.64)^b,c^	1.24 (0.64)	1.27 (0.57)	56.91	<.001	.31
Introjected regulation	3.35 (1.55)^b,c^	1.67 (0.97)^d^	2.69 (1.73)	25.63	<.001	.17
Identified regulation	3.43 (1.48)^b,c^	4.17 (1.23)^d^	5.91 (0.75)	112.57	<.001	.47
Integrated regulation	2.42 (1.18)^c^	2.64 (1.03)^d^	5.38 (0.99)	227.86	<.001	.64
Intrinsic motivation	2.60 (1.31)^b,c^	5.09 (1.22)^d^	6.29 (0.82)	252.28	<.001	.66
Weekly activity score pre-hospitalisation	8.83 (13.76)^b,c^	15.84 (18.15)^d^	22.41 (18.20)	14.47	<.001	.10
Weekly activity score post-discharge	15.86 (15.50)^c^	19.03 (15.59)^d^	32.02 (17.46)	25.66	<.001	.17
Remaining inactive group (*n* = 119)	53 (44.5%)	43 (36.1%)	23 (19.3%)	22.36	<.001	.34
Becoming active group (*n* = 73)	19 (26%)	16 (21.9%)	38 (52.1%)			

Note: Data are given as mean ± SD, % or absolute numbers.

^a^High education comprises vocational baccalaureate diploma, high school diploma, university degree.

^b^*p* < .05 Moderate controlled cluster vs. Moderate autonomous cluster.

^c^*p* < .05 Moderate controlled cluster vs. High autonomous cluster.

^d^*p *< .05 Moderate autonomous cluster vs. High autonomous cluster.

**Figure 2.**
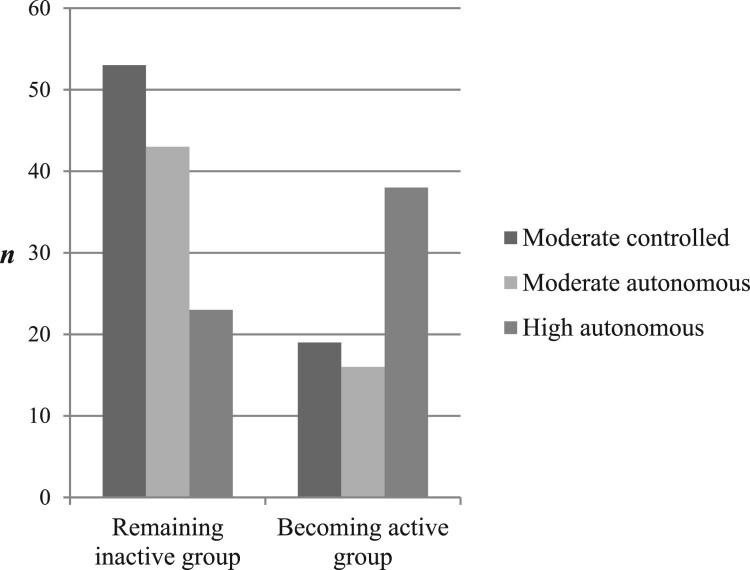
Differences between motivational profiles and exercise behaviour change groups. Moderate-controlled cluster: *n* = 80; Moderate-autonomous cluster: *n* = 78; High-autonomous cluster: *n* = 104.

Regarding the relationship between exercise motivational profiles and exercise change groups, the results showed an association between exercise motivational profiles and exercise change groups: *x*^2^(2) = 22.36, *p* < .001, *V* = .34 (Table 2, Figure 2). Post-hoc tests revealed that significantly more patients who remained inactive belonged to the moderate-autonomous cluster, compared to the high-autonomous cluster (*x*^2^(1) = 14.99, *p* < .001, *V* = .35, Table 2). Additionally, significantly more patients who remained inactive belonged to the moderate-controlled cluster, compared to the high-autonomous cluster (*x*^2^(1) = 17.39, *p* < .001, *V* ^^= .36). Contrary to the above findings, equal amounts of patients who remained inactive or became active belonged to the moderate-autonomous or the moderate-controlled cluster (*x*^2^(1) = 0.09, *p* = .54, *V* ^^= .01).

## Discussion

The purpose of the present study was to investigate how obese patients’ exercise motivational profiles relate to exercise behaviour change from pre-hospitalisation to six months post-discharge. Three distinct profiles emerged over time: the *moderate-controlled*, the *moderate-autonomous* and the *high-autonomous cluster*. While high-autonomous motivation appears to be most favourable regarding prolonged exercising, moderate-autonomous and moderate-controlled motivations also seem to be favourable, and equally so, despite their obvious differences in autonomous regulations.

### Exercise motivational profiles

Obese patients in the *moderate-controlled cluster* partly exercise due to perceived external and internal pressure (i.e. external and introjected regulation). However, this perceived pressure exists alongside some degree of personal valuation (i.e. identified regulation), perhaps implying that introjected-related reasons for exercising are easy to align to other personal values and attitudes when the pressure on the self would otherwise become too intense. Indeed, it might be viewed as a psychological compromise stemming from the innate human tendency to reduced unpleasant feelings (e.g. guilt, shame): instead of being pressurised to comply with norms, patients attempt to align introjected-related reasons to their beliefs and attitudes thereby compensating for the pressure to comply with norms and expectations, in the hope to making the internal sanctions inherent within introjected regulation bearable (i.e. pressure-free and balanced), when exercising (Wasserkampf & Kleinert, 2016). Although the moderate-controlled cluster type has been previously found among inactive individuals (Friederichs et al., 2015), further research is needed to determine exactly how introjected and identified regulations affect each other in obese patients.

Patients in the *moderate-autonomous cluster*, in contrast, understand the importance of exercising and derive pleasure from it, although their identification with exercise is inconsistent and discrepant from other personally essential aspects of life (i.e. low integrated regulation). Accordingly, obese patients belonging to this cluster would not deem themselves to be an exercising person or consider exercising to be an integral part of who they are. Although the treatment might have let patients experience joy, interest and meaningfulness in exercise, it did not encourage patients to sustain exercise behaviour long enough to turn it into an identity-defining or identity-enforcing behavioural standard that needs to be maintained. Despite the enjoyment and valuation of exercise, patients in this profile are not ‘*far*’ into the internalisation process, nor did they adopt exercise behaviour. Given that the present profiles were found in an obese sample, the comparability to other samples is limited. The few studies that have included integrated regulation, however have not reported a profile similar to our moderate-autonomous cluster (similar high-autonomous profiles were found; (Gourlan, Trouilloud, & Boiché, 2016; Miquelon et al., 2016)). The inclusion of integrated regulation therefore proves to be beneficial as it allowed the identification of a different/additional cluster for obese patients.

Finally, obese patients in the *high-autonomous cluster* enjoy exercising as they are acting in accordance with their interests and personal commitments, which results in higher self-satisfaction, as opposed to acting to meet external expectations (Vansteenkiste et al., 2009). As previous research links more self-determined regulations with sustained exercise participation in obese (Silva et al., 2011; Teixeira et al., 2015), this cluster is assumed to be the most favourable in terms of exercise adoption. The high-autonomous cluster has been found in similar manifestations in several studies investigating inactive individuals (Friederichs et al., 2015; Gourlan et al., 2016; Guérin & Fortier, 2012; Miquelon et al., 2016).

### Relationship between motivational profiles and exercise change groups

As expected, most obese patients who became active over time belonged to the high-autonomous cluster. More surprisingly, the moderate-controlled and the moderate-autonomous cluster also appear to be favourable, and equally so, regarding prolonged exercise behaviour change, despite their obvious differences in autonomous regulations. Obese patients’ motivational profiles that are characterised by high-autonomous and relatively low controlled motivation may reflect that the inpatient treatment positively impacted these patients and that this group truly and permanently adopted the treatment’s goal (i.e. becoming more active) as their own. Due to high exercise internalisation (i.e. high scores on identified, integrated and intrinsic motivation), these patients are likely to expend effort to keep exercising beyond six months post-discharge.

The comparison of the moderate-controlled and the moderate-autonomous cluster shows that they do not differ substantially in terms of their *behavioural outcomes* (i.e. exercising), despite of their differences in behavioural regulations. The inpatient treatment thus positively impacted these patients too, however only on a behavioural level. These findings are contradictory to what was hypothesised and at first glance may even be unexpected when considering the theoretical basis of OIT, as more self-determined motivation is clearly linked to more exercise participation behaviour, whilst controlled motivation shows mixed associations (Teixeira, Carraça, Markland, Silva, & Ryan, 2012). Furthermore, previous studies omitting integrated regulation have shown profiles high in identified and intrinsic motivation (i.e. similar to our moderate-autonomous cluster) to be associated with higher exercise participation (Castonguay & Miquelon, 2017; Friederichs et al., 2015; Lindwall et al., 2017), which is contradictory to our results (i.e. moderate-autonomous cluster is strictly speaking the cluster with the least amount (21.9%) of patients who became active). The current findings thus might challenge previous research by suggesting that only ‘*complete*’ autonomous motivation including identified, integrated and intrinsic motivation is favourable regarding long-term exercise persistence. In turn, it thus seems not to matter if patients are moderately controlled or moderately autonomous motivated, as both profiles lead to similar exercise outcomes, though less favourable ones compared to the high-autonomous profile.

Upon further examining both profiles, it becomes apparent that, although the treatment positively impacted patients of both profiles on the behavioural level (i.e. exercise), it did leave both groups with qualitatively distinct motivational profiles, which might affect the patients’ exercise maintenance differently. While patients within the moderate-controlled cluster exercise because they feel they have to, patients within the moderate-autonomous cluster exercise due to the meaningfulness and joy they derive from the activity. Consequently, although both clusters end up with similar behaviour changes, their motivational orientations are quite different. Patients in the moderate-controlled cluster might have developed unrealistic expectations as to the extent to which exercise could facilitate weight loss. As weight loss is a stepwise process, the lack of immediate progress might cause these patients to question the effectiveness and value of exercise as a means to weight loss (Edmunds, Ntoumanis, & Duda, 2007). Obese patients in this cluster are torn between an internal obligation that pushes them to exercise (i.e. introjected regulation) and personal valuation (i.e. identified regulation). The concomitant levels of both regulations might finally prevent the activation of one regulation (i.e. identified regulation) over the other (i.e. introjected regulation), leaving introjected regulation steering behaviour (see Ryan, Koestner, & Deci, 1991). The resulting uncertainty about exercise might impede the integration of exercise into their sense of self. Presumably, patients that are left with moderate levels of introjected regulation, whether or not they also hold moderate levels of identified regulation, are therefore less likely to achieve or maintain an active exercise status, or simply as likely as patients who have lower but nevertheless predominant autonomous motives (i.e. moderate-autonomous cluster; Chamberland & Castonguay, 2016).

In contrast, as exercising feels more self-relevant for patients in the moderate-autonomous cluster, it might be easier for those patients to adhere to exercise and incorporate it into their daily routines. Nonetheless, exercise participation rates are still low in this cluster, possibly highlighting a lack of integrated regulation and therefore no anchorage of exercise into the patients’ self-concept. Accordingly, patient profiles that include intrinsic motivation as well as integrated and identified regulation seem to be more promising regarding exercise behaviour change than autonomous motivated profiles that lack integrated regulation.

### Theoretical implications

From a theoretical perspective, integrated regulation appears to have a special function in the present results as it displays the most noticeable distinction between the high-autonomous and the moderate-autonomous cluster. The function of integrated regulation plausibly stems from the underlying theoretical concepts of internalisation and alignment of behaviour into one’s self. Previous research among active adults has shown that as soon as exercise behaviour is deemed important and becomes integrated into one’s self and aligned with other personal relevant values, norms, and goals, it becomes much more likely that exercise will be engaged in more regularly (Duncan, Hall, Wilson, & Jenny, 2010; Wilson, Rodgers, Loitz, & Scime, 2006) and that it will be sustained in the face of obstacles (Miquelon et al., 2016). The present results complement these findings, suggesting that sustained exercise behaviour, including the associated commitments and organisational challenges, is not dependent on perceived meaningfulness (i.e. identified regulation) or enjoyment (i.e. intrinsic motivation), but rather the integration of the behaviour into the self (i.e. integrated regulation). Motivational profiles including integrated-regulated reasons for exercise might, therefore, provide a ‘*psychological shield*’ for obese patients that best protects against relapses. When exercise is aligned with other important values and needs, behaviour is more stable and anchored within personality, making the behaviour resistant to obstacles (e.g. competing demands, fatigue) that are likely to be encountered post-discharge. This might explain why integrated regulation plays a protective role in exercise maintenance. However, the exact role integrated regulation plays in ensuring lasting behaviour change in obese patients remains questionable, not least because integrated regulation measures are frequently omitted, reducing the reliability of conclusions.

Given OIT’s central, though rigid, distinction between autonomous and controlled motivation, it was expected that both cluster types would be found in the present study. In line with previous research (Friederichs et al., 2015; Lindwall et al., 2017; Miquelon et al., 2016), a high autonomous cluster paired with low controlled motivation scores was clearly identified. However, there was less support for a purely controlled cluster showing high controlled but low autonomous regulations. A lack of a purely controlled profile was also reported in previous research (Lindwall et al., 2017). The absence of a purely controlled cluster might be attributable to social desirability response bias, as participants may feel forced to give responses to items that tap controlled motivation in a way that is not consistent with their personal perceptions and experiences (Wasserkampf & Kleinert, 2016). To avoid social disapproval and feelings of blame, therefore, obese patients may score relatively low to moderate on the controlled items in combination with less than average scores on the more autonomous items, offering a plausible explanation as to why a moderate-controlled cluster was identified and not a purely controlled one.

It could be argued that, in a psychological sense, the response scale of controlled regulations is more restricted compared to autonomous scales, and that z-standardisation might be warranted. Z-standardisation is, however, not without criticism in analyses of subgroups of observations, as it changes, often in undesired ways, the distances between observations and the multivariate distribution of cross-sectional (and longitudinal) data. Moreover, as it is not advisable to first standardise variables within units (e.g. clusters) and then compare mean scores across those units that gave the reference frame for the standardisation (Little, 2013; Moeller, 2015), for these reasons standardisation was not performed. Future research is encouraged to think critically about standardisation or consider alternative scale transformations such as the proportion of maximum scaling (POMS, Little, 2013) or percent of maximum possible (POMP, Cohen, Cohen, Aiken, & West, 1999).

Although a purely controlled profile was not found and the moderate-controlled and moderate-autonomous clusters deviate from the theoretical continuum underpinnings, the profiles still might be ordered along the continuum from higher to lower self-determined motivation. Most similar to intrinsic and high quality motivation would be the high-autonomous cluster, followed by the moderate-autonomous cluster that could be located between most optimal motivation and the middle of the continuum and the moderate-controlled cluster located around the middle of the continuum.

The present profiles thus support the multidimensionality notion of behavioural regulations by reflecting within-person regulation constellations that partly support but also transcend the standard OIT division of being either autonomously or controlled motivated. They thus reflect constellations among autonomous and controlled regulations that are impossible to uncover when analyzing the six regulations individually. Moreover, person-centered approaches are more advantageous in comparison to the RAI. While the RAI assumes that a person is located at one single point on the continuum, (thereby disregarding that this position is derived from multiple regulations and thus multiple locations on the continuum), the relationship between this single RAI-score and outcomes (e.g. exercise) cannot be ascribed to either the ‘autonomous’ or ‘controlled’ part of the RAI-formula (Chemolli & Gagné, 2014). Crucial information for the planning and further development of inpatient-obesity treatments will thus be obscured when using the RAI, leaving practitioners alone in deciding whether to maximise autonomous or minimise controlled regulations (Chemolli & Gagné, 2014). Person-centered approaches, thus offer information that clearly establish what type of treatment to provide; crucial information that cannot be obtained from high, moderate or low scores on the RAI.

As a final theoretical implication it should be stressed that although SDT acknowledges the co-existence of various regulations when regulating behaviour, the theory still needs to specify explanations concerning the different regulation constellations’ underlying mechanisms. In order to better understand certain constellations (e.g. co-existence of introjected and identified regulation in predicting adaptive outcomes), additional theoretical input is thus highly needed.

### Practical implications

The detection of distinct exercise motivational profiles observed 6-months post-discharge provides crucial information for practitioners about long-term exercise maintenance, not only by indicating how behaviour is motivated (by qualitatively distinct constellations of regulations), but also whether and how treatment induced-exercise changes have been sustained over time and for how long patients have expended effort to sustain such outcomes. Based on these retrospective profiles, the inpatient treatment can be rearranged to create more tailored treatments that fully impact patients with different motivational profiles and thus increase the likelihood of behavioural maintenance. As a ‘complete’ autonomous motivational profile appeared most favourable for exercise persistence, and given that integrated regulation might best reflect if exercise has been sustained sufficiently long enough to become an identify-enforcing behavioural standard, a focus on the promotion of integrated regulation should be made a priority in treatment settings (e.g. by helping patients to build an exercise identity (e.g. I’m the families sportsman/sportswoman)).

### Limitations

A major *theoretical* limitation pertains to the exclusive focus on behavioural regulations, which neglects basic psychological needs (i.e. for autonomy, competence, relatedness), as important antecedents of regulations. According to SDT’s theoretical underpinning, the social context (i.e. the exercise context during treatment or thereafter) might have thwarted or supported these needs and consequently influenced how autonomously regulated patients felt towards exercising, contributing to the form of their motivational profiles. Future research is encouraged to consider contextual factors in determining motivational profiles and also to prove that the profiles identified are indeed patterns of associations rather than statistical contortions (i.e. profiles high in autonomous scores should go along with high need satisfaction; Lindwall et al., 2017).

Regarding more *methodological* limitations, the combined design of longitudinal (for exercise) and cross-sectional and retrospective assessments (for exercise behavioural regulations) prevents the inference of causal relationships between exercise motivational profiles and exercise behaviour post-discharge. Specifically, given that motivational profiles were assessed retrospectively six months post-discharge, it is impossible to ascribe changes in exercise behaviour status from pre- to 6-months post-discharge to changes in exercise motivation profiles occurred from pre-hospitalisation to hospital-discharge and to 6-months post-discharge. This approach was chosen, however, firstly, because the inactive group did not have sport and exercise experience prior to admission, which explains why assessing behavioural regulation initially was impossible and secondly, because motivational profiles assessed immediately after discharge would only reflect the patients’ current exercise motivation rather than provide insights into how far patients were able to internalise and sustain treatment outcomes over time. Future research is encouraged to additionally assess motivational profiles across meaningful transition phases (e.g. pre-hospitalisation, discharge and post-discharge), as this would offer valuable information about developmental changes in motivational profiles, which would better explain changes in exercise behaviour.

Regarding the statistical analyses, a major criticism of cluster analysis pertains to the researchers’ subjectivity, which might bias the choice of the final cluster solution (Aldenderfer & Blashfield, 1984). Latent profile analysis (LPA), in comparison to cluster analysis, is more advanced and, as it is a model-based technique, allows greater flexibility regarding model specification and provides different fit indices that aid in the determination of the number of profiles. Nonetheless, LPA has been rarely used so far, making comparability to other studies difficult. Thus, despite LPA’s advantages, cluster analysis was chosen. Although we attempted to validate the current cluster decision and to present level of agreement measures (Cohen’s kappa), the lack of robust statistical indices to make sound decisions regarding the final solution still remains as a limitation. Consequently, the robustness and replicability of the identified clusters should be examined in future studies. Nonetheless, it should be emphasised that specific profiles are likely to reoccur across samples and behaviours (e.g. pure autonomous and controlled but also combined profiles), although the shape/form and the exact number of profiles however depend on the data. Assumptions about replication remain thus questionable. Moreover, the sample reflects an imbalance in gender, a substantial standard deviation of age and a striking difference in psychological disorders between genders, which might have affected the results. The distribution of patients to their respective clusters as well as a potential skewness of the data warrants careful interpretation of findings. The lack of a control group presents an additional weakness, as a control group could have provided additional cases for the cluster analysis.

Furthermore, the sample-specific nature of the present findings limits its generalisability to other populations and/or health behaviours. Nonetheless, it might be assumed that the co-occurrence of introjected and autonomous regulations is observable in all behavioural contexts that allow for the evocation of self-conscious emotions inherent in introjected regulation (i.e. behaviours that are achievement oriented, allow for judgements and lead to by others desired statuses; Martin-Ginis & Leary, 2004) and contexts that allow for need satisfaction (as satisfied needs are important antecedents of successful internalisation and thus autonomous regulations). Eating/weight loss and academic behaviours could represent such behaviours in which similar results might be expected.

## Conclusion

The results of this study indicate that obese patients hold multiple regulations for exercise participation. Thus, considering the full array of behavioural regulations together helps to better understand motivational constellations that go beyond the classification of being autonomous or controlled motivated, something that cannot be accounted for when applying variable-centred approaches or omnibus scores (e.g. RAI). Additionally, the present results shed some light on the under investigated exercise motivational profiles of obese patients in aftercare, therefore providing valuable information and insights into whether and how treatment-induced behavioural changes are sustained over time.

Future studies are warranted to isolate the effect that introjected (by using more refined instruments that distinguish between approach- and avoidance-introjection) and integrated regulation exert on the behaviour change process, which might shed light on the sometimes contradictive relationships between introjection or integrated regulation on exercise or other types of regulations (e.g, identified regulation). In conclusion, the present study complements one of the basic assumptions of OIT that individuals (i.e. obese patients in the present study) are driven (i.e. attracted and distracted) by a variety of reasons when it comes to exercise participation.
